# Vitamin K Antagonists and Cognitive Decline in Older Adults: A 24-Month Follow-Up

**DOI:** 10.3390/nu10060666

**Published:** 2018-05-24

**Authors:** Antoine Brangier, Guylaine Ferland, Yves Rolland, Jennifer Gautier, Catherine Féart, Cedric Annweiler

**Affiliations:** 1 Department of Geriatric Medicine, University Memory Clinic; Research Center on Autonomy and Longevity (CeRAL), University Hospital, F-49933 Angers, France; Antoine.Brangier@chu-angers.fr (A.B.), jegautier@chu-anger.fr (J.G.); 2 Centre de Recherche, Institut Universitaire de Gériatrie de Montréal, Montréal, QC H3W 1W5, Canada; guylaine.ferland@umontreal.ca; 3 Department of Geriatric Medicine, Institut du Vieillissement, University Hospital, INSERM-U1027, F-31000 Toulouse, France; rolland.y@chu-toulouse.fr; 4 Institut de Santé Publique, d’Épidémiologie et de Développement ISPED, Centre INSERM U897-Epidemiologie-Biostatistique, Université Bordeaux, F-33000 Bordeaux, France; Catherine.Feart@isped.u-bordeaux2.fr; 5 Health Faculty and UPRES EA4638, University of Angers, F-49100 Angers, France; 6 Robarts Research Institute, The University of Western Ontario, London, ON N6A 5B7, Canada

**Keywords:** vitamin K antagonist, vitamin K, cognition, executive functions, older adults

## Abstract

Vitamin K participates in brain physiology. This study aimed to determine whether using vitamin K antagonists (VKAs), which interfere with the vitamin K cycle, were (i) cross-sectionally associated with altered cognitive performance, and (ii) independent predictors of cognitive changes in older adults over 24 months. Information was collected on the use of VKAs (i.e., warfarin, acenocoumarol, and fluindione) among 378 geriatric outpatients (mean, 82.3 ± 5.6 years; 60.1% female). Global cognitive performance and executive functions were assessed with Mini-Mental State Examination (MMSE) and Frontal Assessment Battery (FAB) scores, respectively, at baseline and after 12 and 24 months of follow-up. Age, gender, body mass index, mean arterial pressure, disability, gait speed, comorbidities, atrial fibrillation, stroke, carotid artery stenosis, leukoaraiosis grade on computed tomography (CT) scan, psychoactive drugs, antidementia drugs, blood-thinning drugs (i.e., anticoagulants other than VKAs, antiplatelet medications), serum creatinine levels, and vitamin B12 concentrations were considered as potential confounders. Using VKAs was associated with lower (i.e., worse) FAB score at baseline (adjusted β = −2.1, *p* = 0.026), and with a decrease in FAB score after 24 months (adjusted β = −203.6%, *p* = 0.010), but not after 12 months (*p* = 0.659). Using VKAs was not associated with any change in MMSE score at baseline (*p* = 0.655), after 12 months (*p* = 0.603), or after 24 months (*p* = 0.201). In conclusion, we found more severe executive dysfunction at baseline and incident executive decline over 24 months among geriatric patients using VKAs, when compared with their counterparts.

## 1. Introduction

Vitamin K antagonists (VKAs) are the drugs most commonly used in the prophylaxis and treatment of thromboembolic disease in older adults [1,2]. In addition to a role in blood coagulation, vitamin K participates in brain health and function [3] by regulating the synthesis of sphingolipids (a constituent of myelin sheaths and neuronal membranes) [4], and through biological activation of vitamin K-dependent proteins (VKDPs) involved in neuron survival [4]. Furthermore, lower vitamin K intakes have been observed in community-dwelling elders at an early stage of Alzheimer’s disease [5]. The possibility of an adverse impact of VKAs on the brain is strengthened by the finding of abnormalities of the central nervous system (CNS) in newborns exposed to VKAs *in utero* [6], and in older adults regularly using VKAs [7]. Additionally, we recently showed that the use of VKAs was directly associated with cognitive impairment in geriatric patients, even while taking into account the history of atrial fibrillation and stroke [8]. This pilot study was limited by its cross-sectional design, which prevented the establishment of a temporal sequence of events. Thus, a longitudinal follow-up remains necessary to conclusively determine whether or not the use of VKAs precedes cognitive decline. We had the opportunity to address this issue in a longitudinal cohort of older outpatients visiting a university memory clinic. The present study aimed (i) to confirm that the use of VKAs was cross-sectionally associated with cognitive impairment, and (ii) to determine whether the use of VKAs was an independent predictor of cognitive changes among older adults visiting a memory clinic, after 24 months of follow-up.

## 2. Materials and Methods

### 2.1. Study Population

Data were collected from the Alzheimer’s Disease and Related Disorders’ study (MERE study) between December 2008 and March 2013 (ClinicalTrials.gov number: NCT01315704). The MERE study is an observational, unicentric, prospective cohort study designed to examine gait and gait changes with time among older adults visiting the Memory Clinic at Angers University Hospital, France, for a subjective memory complaint. The sampling and data collection procedures have been described elsewhere in detail [9]. In summary, subjective memory complaints were documented using the Subjective Memory Complaints Questionnaire [10], and at least one complaint about memory lapses, learning, episodic memory, working memory, and/or problems concentrating was required before being addressed to the Memory Clinic. The main exclusion criteria were Mini-Mental State Examination (MMSE) score ≤10 [11], inability to walk independently, history of any acute medical illness in the preceding three months, poor vision, inability to understand or answer the study questionnaires, and refusal to participate in research.

Participants received a full medical examination at baseline, which consisted of structured questionnaires, a clinical examination, a neuropsychological assessment, a blood test, and a computed tomography (CT) scan of the brain. When applicable, the same extent of examination was performed again after 12- and/or 24-month follow-up, except for the CT scan.

### 2.2. Dependent Variables: Cognitive Scores

The Mini-Mental State Examination (MMSE) [11] and the Frontal Assessment Battery (FAB) [12] were performed at baseline and during follow-up visits by a trained neuropsychologist without knowledge of the treatments used.

The MMSE score was used to assess the cognitive performance as a whole. The MMSE is a well-established measure of cognitive function in older adults, composed of five sections (orientation, registration, attention and calculation, recall, and language). It shows good test–retest and inter-rater reliability, and performs satisfactorily against more detailed measures of cognitive function [11]. Scores range from 30 (normal) to 0 (impaired).

The FAB score was used to assess the performance of executive functions. The FAB consists of six items, with scores on each item ranging from 0 to 3 (total score range: 0–18, the higher the better). A lower score indicates a more significant degree of executive dysfunction. The six subtests of the FAB explore: similarities (conceptualization), lexical fluency (mental shifting), Luria motor sequences (programming), conflicting instructions (sensitivity to interference), a go-no go test (inhibitory control), and prehension behavior (environmental autonomy). The reliability of the FAB is good to excellent, and its validity has been established [12].

For both cognitive measures, the change in cognitive performance as a percentage (MMSE or FAB) during follow-ups (i.e., after 12 and 24 months) was defined as: delta (D) = [(score at the end − score at the beginning)/(score at the end + score at the beginning)/2)] × 100.

### 2.3. Explanatory Variable: Use of VKAs

Information on the regular use of VKAs was systematically sought by an expert senior physician during standardized face-to-face interviews by questioning patients and their relatives. Participants were asked to bring all medication they were regularly taking to the clinical center. Medical prescriptions and medications themselves were checked. The type of VKA used (i.e., coumarin drugs (warfarin or acenocoumarol) or fluindione) was specified, regardless of indication, length of treatment, or history of international normalized ratio (INR). Continuation of VKA use was checked at every follow-up visit.

### 2.4. Covariables

The following covariables were measured during the assessment at baseline: age; gender; body mass index (BMI); mean arterial pressure (MAP); instrumental activities of daily living (IADL) score; gait speed; number of comorbidities; history of atrial fibrillation; ischemic stroke; carotid artery stenosis; grade of leukoaraiosis; use of psychoactive drugs, antidementia drugs, and other blood-thinning drugs; and serum concentrations of creatinine and vitamin B12.

BMIs were calculated in kg/m^2^, as weight/height^2^. Blood pressures were measured by trained nurses at rest in a quiet environment, according to standardized protocol [13], using a sphygmomanometer placed on the brachial artery with the arm at heart level. MAPs (i.e., average blood pressure over the entire course of the blood pressure cycle) were calculated in mmHg from systolic and diastolic blood pressures (SBPs and DBPs) using the following formula: $\mathrm{MAP}=\frac{SBP+2DBP}{3}$ [13]. Functional autonomy was assessed using IADL scores (/4), and disability was defined as an IADL score of 2 or lower [14]. Gait speed (cm/s) was measured at usual pace, using an electronic portable walkway with pressure sensor pads connected to a computer (GAITRite^®^ Gold walkway, 972 cm long, active electronic surface area—792 × 610 cm, with a total of 29,952 pressure sensors, scanning frequency—60 Hz, software version 3.8, CIR Systems, Havertown, PA, USA). To avoid acceleration and deceleration effects, participants started walking one meter prior to reaching the electronic walkway, and completed their walks one meter beyond it. Evaluation of comorbidities (i.e., diseases lasting at least three months and running a course with minimal change, regardless of etiology) was based on self-report and medical records. Histories of continuous or paroxysmal atrial fibrillation were sought from correspondence with the family physician and patients’ files, by questioning patients or relatives, and with systematic electrocardiograms. Histories of stroke were also sought by questioning patients, family physicians, and patients’ files. Strokes were defined, according to World Health Organization criteria, as rapidly developed signs of focal or global disturbances of cerebral functions lasting longer than 24 h, without apparent nonvascular cause [15]. In cases of clinical suspicion, a CT scan was necessary to confirm the diagnosis. In addition, the use of psychoactive drugs (i.e., benzodiazepines, antidepressants, or neuroleptics), antidementia drugs (i.e., acetycholinesterase inhibitors, memantine, or ginkgo biloba), anticoagulants other than VKAs (i.e., heparin, enoxiparin, tinzaparin, nadroparin, dalteparin, fondaparinux, danaparoid, enoxaparin, or direct oral anticoagulants (DOACs)), and antiplatelet medications (i.e., aspirin, clopidogrel, ticlopidin, dipyridamole) were systematically noted from prescriptions by primary care physicians, and sought by questioning patients and relatives. Grades of leukoaraiosis were evaluated from CT scan images by a single observer (AB) who was blinded to participants’ clinical information, including but not limited to treatments and cognitive performance. Total extents of age-related white matter changes (ARWMC) were measured using the semiquantitative visual rating scale devised by Wahlund and colleagues [16], with scores ranging from 0 (no lesions) to 3 (diffuse involvement of the entire region, with or without involvement of U-fibers). Finally, serum concentrations of creatinine and vitamin B12 were determined using automated standard laboratory methods at the University Hospital of Angers, France.

### 2.5. Statistical Analysis

Participants’ characteristics were summarized using means and standard deviations (SDs), or frequencies and percentages, as appropriate. The sample distribution was checked using the Kolmogorov–Smirnov test. Firstly, comparisons of baseline characteristics, according to the use of VKAs, were performed using the chi-square test or Fisher’s exact test for qualitative variables, and Student’s *t*-test or the nonparametric Mann–Whitney *U* test for quantitative variables, as appropriate. Secondly, multiple linear regressions were used to examine cross-sectional associations of the use of VKAs (independent variable) with MMSE scores and FAB scores at baseline (dependent variables), while adjusting for baseline characteristics. A separate analysis was performed for each cognitive score. Thirdly, we examined changes in MMSE and FAB scores after 12 and 24 months of follow-up according to the use of VKAs. Finally, associations of the use of VKAs at baseline (independent variable) with MMSE and FAB scores after 12 and 24 months of follow-up (dependent variables) were examined with separate linear regression models, adjusted for all covariables. In this study, *p*-values < 0.05 were considered statistically significant. All statistical analyses were performed using Statistical Package for the Social Sciences (SPSS, v19.0, IBM Corporation, Chicago, IL, USA).

### 2.6. Ethics

Participants were only included after having given their informed consent for research. The study was conducted in accordance with the ethical standards set forth in the Declaration of Helsinki (1983). The study protocol was approved by the local ethical committee (2009/15).

## 3. Results

This study recruited 378 participants (mean ± standard deviation, 82.3 ± 5.6years, 60.1% female). Among them, 46 participants (12.2%) used VKAs. The mean MMSE score was 19.8 ± 4.8, and the mean FAB score was 11.8 ± 3.2.

Table 1 indicates the baseline participant characteristics, according to their use of VKAs. Participants using VKAs had similar MMSE scores when compared with those who did not use VKAs (20.0 ± 3.9 versus 19.8 ± 4.9, respectively, *p* = 0.778), but lower (i.e., worse) FAB scores (10.3 ± 3.6 versus 12.0 ± 3.0, respectively, *p* = 0.006). They also exhibited higher BMIs (*p* < 0.001) and a greater number of comorbidities (*p* = 0.005), including more frequent atrial fibrillation (*p* < 0.001) and stroke (*p* = 0.001). Finally, they used antiplatelet medications less often (*p* < 0.001). There were no significant differences across both groups for the remaining clinical characteristics (Table 1).

Table 2 shows multiple linear regressions for MMSE and FAB scores at baseline, with the use of VKAs and baseline characteristics as explanatory variables. The use of VKAs was not associated with MMSE scores (fully adjusted β = 0.51, *p* = 0.655), but was significantly and negatively associated with FAB scores (fully adjusted β = −2.12, *p* = 0.026). Gait speed and the use of antidementia drugs were associated both with MMSE scores (β = 0.07 with *p* < 0.001, and β = −3.63 with *p* < 0.001, respectively) and FAB scores (β = 0.05 with *p* < 0.001, and β = −1.57 with *p* = 0.001, respectively). BMI and disability were also associated with MMSE scores (β = 0.19 with *p* = 0.011, and β = −2.95 with *p* < 0.001, respectively), but not with FAB scores (Table 2).

Among the 378 patients enrolled in the MERE analysis, 170 (45.0%) were re-examined after 12 months, and 58 (15.3%) were re-examined after 24 months, as part of their routine follow-up. The characteristics of those who were followed-up with at 12 and 24 months were very similar to their counterparts, except for FAB scores at baseline, which were higher among those followed-up with at 12 months (12.24 ± 3.07 versus 11.44 ± 3.17 respectively, *p* = 0.045) and those followed-up with at 24 months (13.62 ± 2.93 versus 11.47 ± 3.08 respectively, *p* < 0.001). There were no differences in MMSE scores at baseline when comparing those followed-up with at 12 months (20.2 ± 4.9 versus 19.5 ± 4.6 at baseline, *p* = 0.162), and at 24 months (20.29 ± 4.60 versus 19.76 ± 4.79 at baseline, *p* = 0.430) to others.

**Figure 1:**
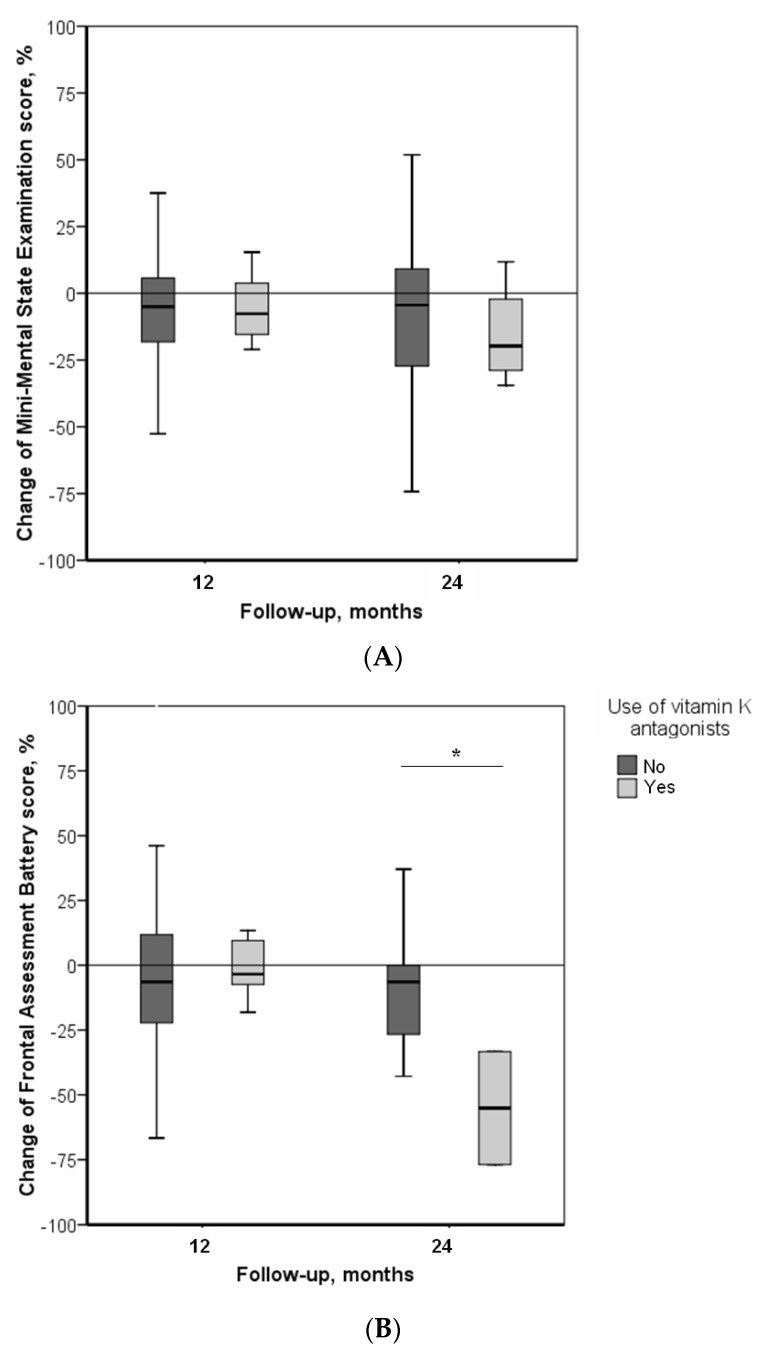
Changes in cognitive scores during follow-up, according to use of vitamin K antagonists ^†^. (**A**) Changes in Mini-Mental State Examination scores; (**B**) Changes in Frontal Assessment Battery scores during follow-up. *: *p* = 0.023 (two-tailed). ^†^: *n* = 170 followed-up at 12 months; *n* = 58 followed-up at 24 months.

Figure 1 illustrates the changes in MMSE and FAB scores after 12 and 24 months of follow-up according to the use of VKAs. Regarding change in global cognitive performance, we found a decrease (i.e., worsening) in MMSE scores during follow-ups, with Δ_MMSE_ = −6.6 ± 20.6% after 12 months of follow-up, and Δ_MMSE_ = −12.9 ± 32.1% after 24 months. There was no difference, according to the use of VKAs, after 12 months (Δ_MMSE_ = −9.4 ± 20.1% among VKA users versus Δ_MMSE_ = −6.3 ± 20.7% among non-users, *p* = 0.553), or after 24 months (Δ_MMSE_ = −15.5 ± 19.7% among VKA users versus Δ_MMSE_ = −12.7 ± 32.9% among non-users, *p* = 0.864). Regarding change in executive functions, the results showed a decrease (i.e., worsening) in FAB scores during follow-ups, with Δ_FAB_ = −4.2 ± 29.3% after 12 months of follow-up, and Δ_FAB_ = −9.0 ± 30.8% after 24 months. Although we found no differences in Δ_FAB_ after 12 months according to the use of VKAs (Δ_FAB_ = −1.6 ± 11.7% among VKA users versus Δ_FAB_ = −4.5 ± 30.7% among non-users, *p* = 0.820), the group using VKAs exhibited a more significant worsening of executive functions after 24 months of follow-up (Δ_FAB_ = −55.5 ± 1% versus Δ_FAB_ = −4.6 ± 27.6%, *p* = 0.023).

Finally, Table 3 shows that the use of VKAs was negatively associated with Δ_FAB_ after 24 months of follow-up (β = −203.6 with *p* = 0.010 after adjustment for all baseline characteristics, including baseline FAB scores), but not after 12 months (*p* = 0.659). In contrast, the use of VKAs was not associated with Δ_MMSE_, neither after 12 months (*p* = 0.603) nor after 24 months (*p* = 0.201). Additionally, there were no interactions between the use of VKAs and other drugs that could significantly alter these results (data not shown).

## 4. Discussion

The main finding of this prospective study of older outpatients with subjective memory complaints is that, irrespective of the studied potential confounders, the use of VKAs was associated with lower (i.e., worse) scores of executive functions at baseline, and with more significant worsening of executive functions after 24 months of follow-up. In contrast, global cognitive performance, assessed with the MMSE score, did not differ according to the use of VKAs.

Few previous studies have examined the relationship between the use of VKAs and cognition. In rodents, some co-authors of our group have reported that the use of warfarin was detrimental to spatial learning performance [17]. In humans, a recent cross-sectional observational study showed that the use of VKAs was associated with the impairment of global cognitive performance in 267 geriatric patients [8]. Similarly, a previous longitudinal observational study of 218 adults reported that, contrary to the assumption made by the authors, the use of warfarin was not associated with a reduced risk of dementia after 3 years of follow-up [18]. Finally, in an earlier trial testing the potential of blood-thinning drugs to prevent cognitive decline, the authors found in 424 adults that, when compared with those using aspirin or placebo, the participants who had used VKAs for at least five years exhibited worse performance in Wechsler Adult Intelligence Scale-III (WAIS-III) similarities (i.e., 19 items exploring concept formation and abstract reasoning, both of which are executive functions) [19]. None of these previous studies examined changes in cognitive scores over time according to the regular use of VKAs. Consequently, our results provide additional and novel evidence on the nature of this relationship, by showing that the use of VKAs is associated with executive dysfunction in older adults, and precedes greater decline in executive functions over 24 months.

The mechanism linking the use of VKAs with cognitive disorders is not fully elucidated. On one hand, the association may be explained by conditions warranting the use of VKAs, such as atrial fibrillation and strokes, which may in turn lead to greater occurrences of cognitive decline [20]. However, this first assumption was unlikely here since the association between VKAs and cognitive disorders persisted after adjustment for these conditions. On the other hand, our results may indicate a specific effect of VKAs on cognition, independent of underlying conditions and the effect of anticoagulants. VKAs interfere with the vitamin K cycle, decreasing availability of the active form of vitamin K (hydroquinone) in the body [1], including in the brain [21]. Basic research supports a role for vitamin K in the brain. It regulates the biological activation of two VKDPs, namely Gas6 (growth arrest-specific gene 6) and protein S [3,4]. Gas6 is involved in chemotaxis, mitogenesis, cell growth, and myelination, and has further been shown to rescue cortical neurons from amyloid-β-induced apoptosis, a hallmark of Alzheimer’s disease (AD) [22]. Protein S offers neuronal protection during ischemic/hypoxic injury [23]. Additionally, vitamin K regulates the metabolism of sphingolipids, which are key players in neuronal proliferation, differentiation, senescence, cell–cell interaction, and transformation [3,4]. Alterations in sphingolipid metabolism may also be involved in neurodegenerative disorders such as AD [4]. Importantly, the use of VKAs has been demonstrated to alter the sphingolipid profile [24]. This suggests that less available vitamin K due to VKAs may lead to altered neuronal function and survival, and subsequently to a greater risk of cognitive decline.

Specifically, we found that executive functions (i.e., FAB scores) were inversely associated with the use of VKAs. Executive functions refer to a heterogeneous set of high-level processes that control and regulate other abilities and behaviors [25]. From a neuroanatomical point of view, executive functions are based on the integrity of frontal-subcortical circuits [25], and a lesion in these circuits, whether atrophic or ischemic, results in impairment of executive functions. Thus, the finding of executive dysfunction in the case of VKAs use may be explained by the loss of vitamin K protective action in the subcortical and frontal brain structures [4]. In contrast, we found no association between the use of VKAs and global cognitive performance (i.e., MMSE scores), even if there was a trend of greater decline in MMSE scores after 24 months of treatment in the group using VKAs, when compared with non-users (Figure 1A). At first sight, this seems to contradict one previous study that reported that people using VKAs had a greater prevalence of impaired global cognitive performance [8]. Inconsistencies could be explained by the fact that, unlike the previous study, we used MMSE score here as a quantitative variable rather than as a dichotomous categorical variable. The latter method provides statistically robust results, allowing us to evaluate numerical changes in cognitive scores during follow-ups. 

The strengths of the present study include the originality of the research question on a drug used in clinical routine, the use of a longitudinal prospective design with an intermediate mid-term evaluation and a final long-term evaluation allowing the examination of cognitive changes over time [26], the detailed description of participant characteristics allowing the use of regression models to measure adjusted associations, and the standardized collection of data from a single research center. Regardless, our study also has some limitations. Firstly, the study cohort was restricted to a limited number of geriatric outpatients, especially at 24 months, which led to large standard errors and confidence intervals for the coefficients of regression, and likely made our sample unrepresentative of the population of all seniors. The MERE study is a pragmatic study on real-life data. As a consequence, some patients were not followed-up with at 12 and 24 months, either because they did not require any particular follow-ups as part of routine care, or because they did not wish to continue their follow-ups, or because their follow-ups were organized at another pace, or because they chose to be followed-up with in another structure. Either way, analysis of their characteristics showed that the groups that were followed-up with and those who were not were very similar at baseline. The main exception was the baseline FAB score, which was statistically higher among those who subsequently had follow-ups, although this small difference should not be considered as clinically relevant. In addition, it is notable that the prevalence of the use of VKAs in our study (12.2%) was similar to that reported by the National Agency of Drug Safety and Health Products in people aged 65 years and older (13.3%) [2], which confirms that this real life data remained valid and relevant, despite the small sample size. Secondly, the MERE study was not initially built to meet the objective of this post-hoc analysis. Thus, some important data were not available and could not be taken into account in the analyses, such as length of VKA treatment, possible history of VKA use before baseline assessment, or history of INR. Thirdly, although we were able to control for important characteristics likely to modify this association, it would have been valuable to consider additional covariables, such as serum phylloquinone concentration, other medications taken, education level, or changes in covariables during follow-up. Fourthly, the use of MMSE and FAB scores may also limit the scope of our results. The MMSE is a screening tool with risks of ceiling effects and test–retest effects, whose clinical utility remains debated, despite recent neuroimaging data evidencing strong correlations with brain health and structure [27]. In addition, the FAB is a composite score exploring executive functioning as a whole. Thus, in future research, it would be contributory to examine the specific effects of VKA use on various subdomains of executive functions, with dedicated subscores.

## 5. Conclusions

We were able to show more frequent and severe executive decline associated with the use of VKAs among geriatric outpatients. This question is all the more crucial because the use of VKAs is widespread in older adults. Clinical trials are now warranted to explore the effect on cognition of the use of VKAs in large samples of adults, against that of DOACs, whose indications are similar, but whose mechanism does not interfere with vitamin K [28].

## Figures and Tables

**Table 1:** Baseline characteristics of 378 participants, according to use of vitamin K antagonists.

	Total Cohort (*n* = 378)	Use Vitamin K Antagonists		*p*-Value
		No (*n* = 332)	Yes (*n* = 46)	
**Demographic measures**				
Age, years	82.32 ± 5.60	82.11 ± 5.79	83.81 ± 3.63	0.160
Female gender, *n* (%)	227 (60.1)	202 (60.8)	25 (54.3)	0.399
**Clinical measures**				
Body mass index, kg/m^2^	27.99 ± 18.30	27.71 ± 19.49	29.91 ± 5.35	<0.001
Mean arterial pressure, mmHg	100.12 ± 12.03	99.74 ± 12.09	102.61 ± 11.45	0.196
IADL score, /4	1.94 ± 1.40	1.94 ± 1.44	1.91 ± 1.11	0.972
Gait speed, cm/s	71.56 ± 24.49	72.56 ± 24.53	65.11 ± 23.60	0.094
Number of comorbidities	3.58 ± 2.12	3.46 ± 2.03	4.51 ± 2.53	0.005
Atrial fibrillation, *n* (%)	56 (14.8)	19 (5.7)	37 (80.4)	<0.001
Stroke, *n* (%)	42 (11.1)	30 (9.0)	12 (26.1)	0.001
ARWMC score, /3	0.68 ± 0.98	0.68 ± 0.98	0.70 ± 1.01	0.904
Carotid artery stenosis, *n* (%)	23 (6.1)	20 (6.0)	3 (6.5)	0.751
MMSE score, /30	19.84 ± 4.76	19.82 ± 4.87	19.96 ± 3.90	0.778
FAB score *, /18	11.78 ± 3.15	12.01 ± 3.01	10.29 ± 3.59	0.006
Use of psychoactive drugs, *n* (%)	223 (59.0)	201 (61.7)	22 (47.8)	0.073
Use of antidementia drugs, *n* (%)	198 (52.4)	172 (51.8)	26 (56.5)	0.548
**Serum measures**				
Creatinine concentration, µmol/L	86.03 ± 48.26	85.34 ± 50.60	91.12 ± 24.86	0.049
Vitamin B12 concentration, ng/L	449.13 ± 231.65	448.06 ± 236.81	457.18 ± 191.59	0.483
**Use of blood-thinning drugs**				
Total, *n* (%)	175 (46.3)	129 (38.9)	46 (100.0)	<0.001
Vitamin K antagonists, *n* (%)	46 (12.2)	-	-	-
Heparin, *n* (%)	4 (1.1)	4 (1.2)	0 (0.0)	1.000
Direct oral anticoagulants, *n* (%)	0 (0.0)	0 (0.0)	0 (0.0)	-
Antiplatelet medications, *n* (%)	129 (34.1)	126 (38.0)	3 (6.5)	<0.001

Data presented as mean ± standard deviation when applicable. ARWMC: age-related white matter changes; FAB: Frontal Assessment Battery; IADL: instrumental activities of daily living; MMSE: Mini-Mental State Examination; *: *n* = 253.

**Table 2:** Fully adjusted linear regressions examining the association between use of vitamin K antagonists and cognitive scores at baseline.

	Cognitive Scores					
	MMSE Score (*n* = 378)			FAB Score (*n* = 253)		
	β	[95% CI]	*p*-Value	β	[95% CI]	*p*-Value
Use of vitamin K antagonists	0.51	[−2.38, 23.26]	0.655	−2.12	[−3.99, −0.25]	0.026
Age	0.05	[−1.75, 2.77]	0.443	0.02	[−0.07, 0.11]	0.635
Female gender	0.80	[−0.07, 0.16]	0.228	0.59	[−0.40, 1.59]	0.242
Body mass index	0.19	[0.04, 0.34]	0.011	−0.03	[−0.15, 0.09]	0.649
Mean arterial pressure	−0.01	[−0.06, 0.03]	0.564	0.002	[−0.04, 0.04]	0.939
Number of comorbidities	0.08	[−0.20, 0.35]	0.578	−0.03	[−0.25, 0.20]	0.823
Disability *	−2.95	[−4.35, −1.55]	<0.001	−0.46	[−1.52, 0.60]	0.393
Gait Speed	0.07	[0.04, 0.10]	<0.001	0.05	[0.03, 0.07]	<0.001
Atrial fibrillation	−0.38	[−2.46, 1.71]	0.722	1.12	[−0.67, 2.92]	0.219
Stroke	0.60	[−1.46, 2.66]	0.566	1.03	[−0.64, 2.69]	0.225
ARWMC score	−0.04	[−0.60, 0.53]	0.897	−0.35	[−0.80, 0.09]	0.115
Carotid artery stenosis	−1.59	[−3.91, 0.73]	0.178	−0.79	[−2.63, 1.06]	0.402
Use of psychoactive drugs	−0.08	[−1.27, 1.11]	0.898	−0.66	[−1.57, 0.25]	0.156
Use of antidementia drugs	−3.63	[−4.79, −2.48]	<0.001	−1.57	[−2.47, −0.67]	0.001
Creatinine concentration	−0.001	[−0.02, 0.02]	0.961	−0.001	[−0.02, 0.02]	0.915
Vitamin B12 concentration	0.00	[−0.00, 0.00]	0.830	0.00	[−0.00, 0.00]	0.836
Use of antiplatelet medications	1.07	[−0.24, 2.38]	0.109	0.02	[−1.01, 1.06]	0.963

β: coefficient of regression corresponding to changes in cognitive score; CI: confidence interval; FAB: Frontal Assessment Battery; MMSE: Mini-Mental State Examination; *: instrumental activities of daily living score (/4) of 2 or lower.

**Table 3:** Fully adjusted linear regressions examining the association between the use of vitamin K antagonists (independent variable) and changes in cognitive scores, as percentages, during follow-up (dependent variables).

	Use Vitamin K Antagonists		
	β	[95% CI]	*p*-Value
Change in MMSE score *			
After 12 months of follow-up	−4.38	[−21.08, 12.32]	0.603
After 24 months of follow-up	45.21	[−26.35, 116.78]	0.201
**Change in FAB score ^†^**			
After 12 months of follow-up	14.35	[−53.54, 82.24]	0.659
After 24 months of follow-up	−203.57	[−246.14, −161.00]	**0.010**

β: coefficient of regression corresponding to changes in cognitive score; CI: confidence interval; FAB: Frontal Assessment Battery; MMSE: Mini-Mental State Examination; *: adjusted for age, gender, body mass index, mean arterial pressure, baseline MMSE score, number of comorbidities, atrial fibrillation, stroke, age-related white matter changes score, carotid artery stenosis, use of psychoactive drugs, use of antidementia drugs, use of antiplatelet medications, and serum concentrations of creatinine and vitamin B12; ^†^: adjusted for age, gender, body mass index, mean arterial pressure, baseline FAB score, number of comorbidities, disability, gait speed, atrial fibrillation, stroke, age-related white matter changes score, carotid artery stenosis, use of psychoactive drugs, use of antidementia drugs, use of antiplatelet medications, and serum concentrations of creatinine and vitamin B12; β significance (i.e., *p*-value < 0.05) indicated in bold.

## References

[1] Ansell J., Hirsh J., Hylek E., Jacobson A., Crowther M., Palareti G. (2008). Pharmacology and management of the vitamin K antagonists: American College of Chest Physicians evidence-based clinical practice guidelines. Chest.

[2] French National Security Agency of Medicines and Health Products (ANSM) Anticoagulants in France in 2012: Inventory and Monitoring. http://ansm.sante.fr/var/ansm_site/storage/original/application/901e9c291a545dff52c0b41365c0d6e2.pdf.

[3] Ferland G. (2012). Vitamin K, an emerging nutrient in brain function. Biofactors.

[4] Ferland G. (2012). Vitamin K and the nervous system: An overview of its actions. Adv. Nutr..

[5] Presse N., Shatenstein B., Kergoat M.J., Ferland G. (2008). Low vitamin K intakes in community-dwelling elders at an early stage of Alzheimer’s disease. J. Am. Diet. Assoc..

[6] Hall J.G., Pauli R.M., Wilson K.M. (1980). Maternal and fetal sequelae of anticoagulation during pregnancy. Am. J. Med..

[7] Annweiler C., Denis S., Duval G., Bartha R., Beauchet O. (2015). Use of Vitamin K Antagonists and Brain Volumetry in Older Adults: Preliminary Results From the GAIT Study. J. Am. Geriatr. Soc..

[8] Annweiler C., Ferland G., Barberger-Gateau P., Brangier A., Rolland Y., Beauchet O. (2015). Vitamin K antagonists and cognitive impairment: Results from a cross-sectional pilot study among geriatric patients. J. Gerontol. A Biol. Sci. Med. Sci..

[9] Beauchet O., Launay C.P., Allali G., Watfa G., Gallouj K., Herrmann F.R., Annweiler C. (2013). Anti-dementia drugs and changes in gait: A pre-post quasi-experimental pilot study. BMC Neurol..

[10] Vannier-Nitenberg C., Dauphinot V., Bongue B., Sass C., Rouch I., Beauchet O., Krolak-Salmon P., Fantino B. (2013). Early detection of memory impairment in people over 65 years old consulting at Health Examination Centers for the French health insurance: The EVATEM protocol. BMC Geriatr..

[11] Folstein M.F., Folstein S.E., McHugh P.R. (1975). Mini-mental state: A practical method for grading the cognitive state of patients for the clinician. J. Psychiatr. Res..

[12] Dubois B., Slachevsky A., Litvan I., Pillon B.F.A.B. (2000). The FAB: A frontal assessment battery at bedside. Neurology.

[13] Pickering T.G., Hall J.E., Appel L.J., Falkner B.E., Graves J., Hill M.N., Jones D.W., Kurtz T., Sheps S.G., Roccella E.J. (2005). Recommendations for blood pressure measurment in humans and experimental animals: Part 1: Blood pressure measurement in humans: A statement for professionals from the Subcommittee of Professional and Public Education of the American Heart Association Council on High Blood Pressure Research. Circulation.

[14] Pérès K., Chrysostome V., Fabrigoule C., Orgogozo J.M., Dartigues J.F., Barberger-Gateau P. (2006). Restriction in complex activities of daily living in MCI: Impact on outcome. Neurology.

[15] Hatano S. (1976). Experience from a multicentre stroke register: A preliminary report. Bull. World Health Organ..

[16] Wahlund L.O., Barkhof F., Fazekas F., Bronge L., Augustin M., Sjögren M., Wallin A., Ader H., Leys D., Pantoni L. (2001). A new rating scale for age-related white matter changes applicable to MRI and CT. Stroke.

[17] Tamadon-Nejad S. Warfarin-Induced Vitamin K Deficiency Is Associated with Cognitive and Behavioral Perturbations, and Alterations in Brain Sphingolipids in Rats. Université de Montréal 2013.

[18] Barber M., Tait R.C., Scott J., Rumley A., Lowe G.D.O., Stott D.J. (2004). Dementia in subjects with atrial fibrillation: Hemostatic function and the role of anticoagulation. J. Thromb. Haemost..

[19] Richards M., Meade T.W., Peart S., Brennan P.J., Mann A.H. (1997). Is there any evidence for a protective effect of antithrombotic medication on cognitive function in men at risk of cardiovascular disease? Some preliminary findings. J. Neurol. Neurosurg. Psychiatry.

[20] Kalantarian S., Sternd T., Mansour M., Ruskin J.N. (2013). Cognitive impairment associated with atrial fibrillation. A meta-analysis. Ann. Intern. Med..

[21] Thijssen H.H., Drittij-Reijnders M.J. (1994). Vitamin K distribution in rat tissues: Dietary phylloquinone is a source of tissue menaquinone-4. Br. J. Nutr..

[22] Yagami T., Ueda K., Asakura K., Sakaeda T., Nakazato H., Kuroda T., Hata S., Sakaguchi G., Itoh N., Nakano T. (2002). Gas6 rescues cortical neurons from amyloid beta protein-induced apoptosis. Neuropharmacology.

[23] Liu D., Guo H., Griffin J.H., Fernandez J.A., Zlokovic B.V. (2003). Protein S confers neuronal protection during ischemic/hypoxic injury in mice. Circulation.

[24] Sundaram K.S., Lev M. (1988). Warfarin administration reduces synthesis of sulfatides and other sphingolipids in mouse brain. J. Lipid Res..

[25] Godefroy O., Jeannerod M., Allain P., Le Gall D. (2008). Frontal lobe, executive functions and cognitive control. Rev. Neurol..

[26] Truffinet P., Bordet R., Menard J. (2009). Relevance of the evaluation criteria used in clinical trials for Alzheimer’s disease. Therapie.

[27] Dinomais M., Celle S., Duval G.T., Roche F., Henni S., Bartha R., Beauchet O., Annweiler C. (2016). Anatomic Correlation of the Mini-Mental State Examination: A Voxel-Based Morphometric Study in Older Adults. PLoS ONE.

[28] Mousa S.A. (2010). Novel anticoagulant therapy: Principle and practice. Methods Mol. Biol..

